# Periorbital necrotizing soft tissue infection due to *Streptococcus pyogenes* with streptococcal toxic shock syndrome: successful early structured multidisciplinary management

**DOI:** 10.1186/s12348-026-00589-8

**Published:** 2026-05-11

**Authors:** Shigeko Yashiro, Kento Inoue, Yuki Kitagawa, Kei Yamamoto, Masako Nagahara, Phung Vu, Mugen Ujiie, Miyuki Nagahara, Norio Ohmagari

**Affiliations:** 1 Department of Ophthalmology, National Center for Global Health and Medicine, Japan Institute for Health Security, 1-21-1 Toyama, Shinjuku-ku, Tokyo, 162-8655 Japan; 2 Disease Control and Prevention Center, National Center for Global Health and Medicine, Japan Institute for Health Security, Tokyo, Japan; 3 Department of Nephrology, Juntendo University, Tokyo, Japan; 4 Department of Ophthalmology, The Graduate School of Medicine and Faculty of Medicine, The University of Tokyo, Tokyo, Japan; 5 Department of Ophthalmology, Princess Alexandra Hospital, Brisbane, Australia; 6 Global Outbreak Intelligence, Capacity Building and Deployment Coordination Center, Disease Control and Prevention Center, National Center for Global Health and Medicine, Tokyo, Japan; 7 WHO Collaborating Centre for Prevention, Preparedness and Response to Emerging Infectious Diseases, Disease Control and Prevention Center, National Center for Global Health and Medicine, Tokyo, Japan

**Keywords:** Periorbital necrotizing soft tissue infection, *Streptococcus pyogenes*, Streptococcal toxic shock syndrome, Surgical debridement, Multidisciplinary management

## Abstract

Periorbital necrotizing soft tissue infections (NSTIs) are rare but potentially life-threatening, particularly when complicated by streptococcal toxic shock syndrome (STSS). Rapid systemic deterioration can occur, even in previously healthy individuals. We report a case of a 57-year-old woman who developed periorbital NSTI, complicated by STSS, secondary to *Streptococcus pyogenes*. Emergency eyelid debridement was performed within 24 h of symptom onset, followed by intensive care management and empirical broad-spectrum intravenous antibiotics, which were subsequently adjusted to pathogen-directed therapy. Localized recurrence on hospital day 6 was treated with repeated, limited debridement and adjunctive, low-concentration antiseptic irrigation. The patient recovered from the STSS. Progressive black necrosis did not occur; visual function was preserved, reconstructive surgery was unnecessary, and complete cosmetic recovery was achieved within eight months. This case underscores that STSS may develop rapidly, even in healthy individuals, and highlights the importance of early, structured, and multidisciplinary management.

## Introduction

Necrotizing soft tissue infections (NSTIs) are rapidly progressive bacterial infections characterized by extensive necrosis of the skin, subcutaneous tissue, and fascia [1, 2]. They are commonly classified as type I (polymicrobial) or type II (monomicrobial), with the latter most frequently caused by group A β-hemolytic streptococci, particularly *Streptococcus pyogenes* [1]. Reported mortality rates for NSTIs range from approximately 20–30%, and may be substantially higher in cases complicated by streptococcal toxic shock syndrome (STSS), a toxin-mediated systemic condition associated with severe hemodynamic instability [1, 2].

Periorbital NSTIs are uncommon, likely due to the rich vascular supply of the eyelids [3–5]. Reported mortality rates in this anatomical region are lower than those observed in other sites, ranging from approximately 3–12% [3–6]. Nevertheless, rapid local progression and systemic deterioration can occur, even in individuals without underlying comorbidities [3, 4]. Extension beyond the eyelid may result in visual loss, orbital complications, the need for reconstructive surgery, or death [3–5, 7]. Previous reports have demonstrated that selected cases of eyelid necrotizing fasciitis can be managed with intravenous antimicrobial therapy and limited surgical debridement [3, 8]. However, cases complicated by STSS that achieve preservation of visual function and favorable cosmetic outcomes without extensive reconstruction remain relatively uncommon [4, 7, 9, 10].

We report a case of periorbital NSTI caused by *Streptococcus pyogenes* in a previously healthy patient who developed STSS and recovered following early, structured, and multidisciplinary management guided by carefully directed surgical intervention, preserving both function and cosmesis.

## Case

A previously healthy 57-year-old woman presented in Japan to a local ophthalmologist with mild erythema of the right upper eyelid, which progressed to marked swelling, blister formation, and severe pain within 24 h. The patient was referred to our institution for evaluation and management of suspected periorbital cellulitis.

**Fig. 1:**
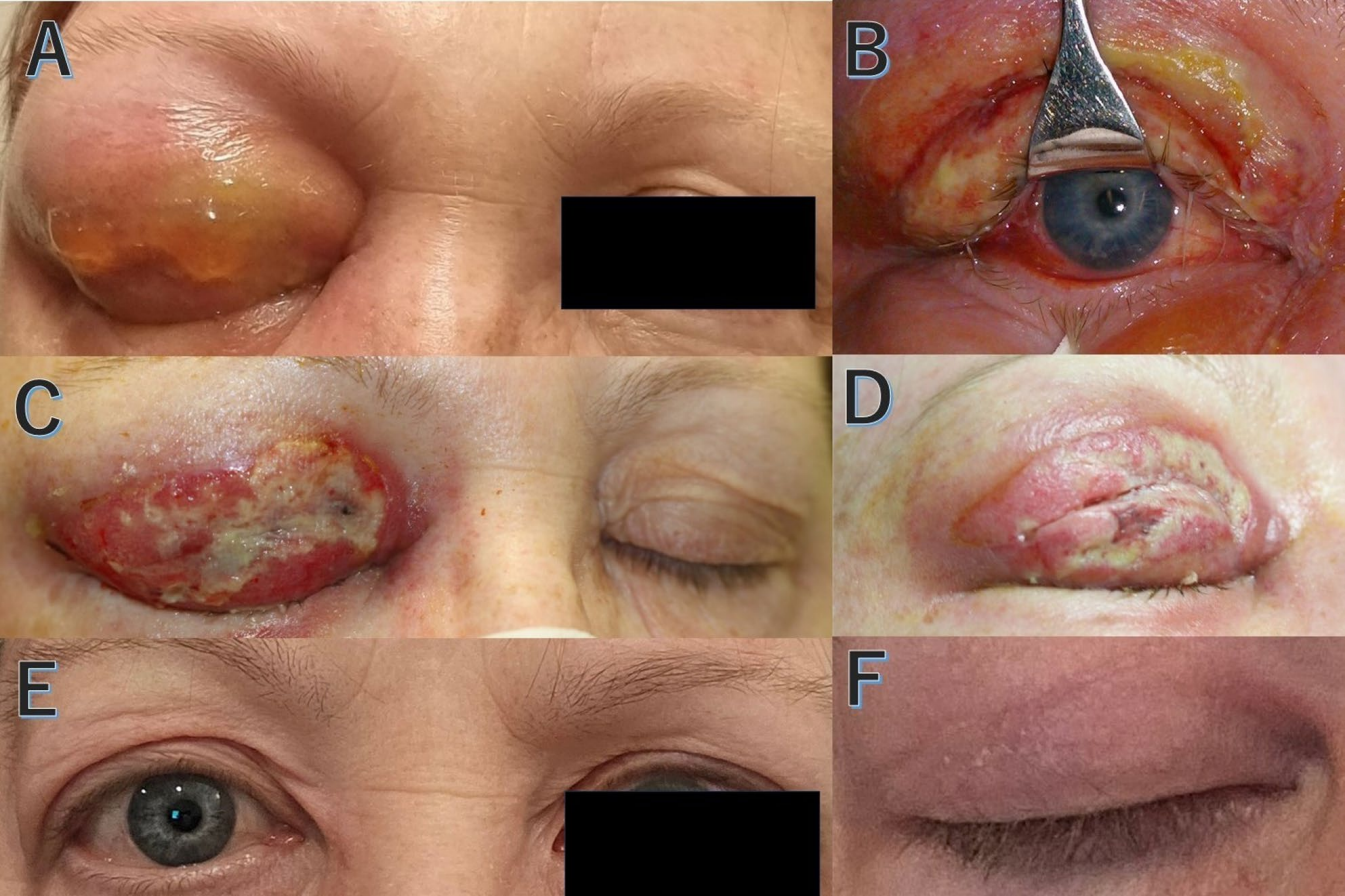
Clinical course of periorbital necrotizing soft tissue infection. (**A**) Day 1 (initial presentation): Erythema with localized skin bullae on the right upper eyelid, prompting urgent clinical assessment. (**B**) Day 3 (two days after debridement): No progression of conjunctival hyperemia or black necrosis following the early surgical intervention. (**C**) Day 6 (transfer to the general ward): Recurrent eyelid swelling with increased ocular discharge necessitating close reassessment. (**D**) Day 9 (discharge): Clinical improvement without residual black necrosis. (**E**, **F**) Month 8: Complete recovery without noticeable asymmetry compared to the contralateral eyelid

Visual acuity could not be assessed owing to the patient ’s inability to open her eyelids secondary to severe edema. The conjunctiva exhibited moderate hyperemia and yellowish discharge. Diffuse erythema with indistinct borders, edema extending beyond the clinically apparent erythema, and localized cutaneous bullae were observed on the right eyelid (Fig. 1A). There was no history of penetrating trauma or recent surgical intervention; however, the swelling extended toward the nasal bridge and temporal region.

**Fig. 2:**
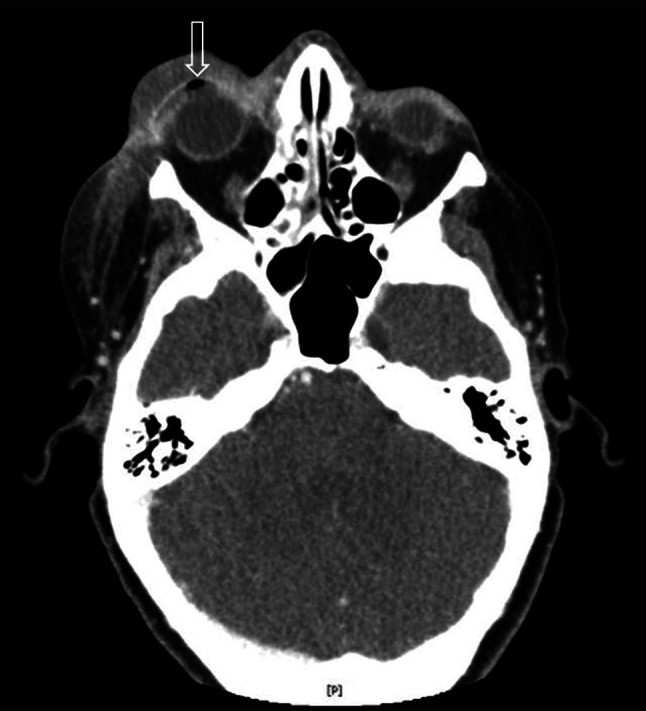
Non-contrast computed tomography at the initial presentation. Bilateral ethmoid sinus mucosal thickening and diffuse preseptal soft tissue swelling are observed, with enlarged right cervical lymph nodes extending from the periorbital to the anterior temporal region. No intraorbital abscess, postseptal extension, or intraocular abnormality is identified. An apparent gaseous shadow over the cornea (arrow) initially raised concerns regarding a gas-forming infection but was subsequently interpreted as trapped air related to severe eyelid edema rather than true soft-tissue gas

Non-contrast head computed tomography (CT) revealed bilateral ethmoid sinus mucosal thickening and diffuse preseptal soft tissue swelling with homogeneous density. Enlarged right cervical lymph nodes extended from the periorbital region to the anterior temporal area (Fig. 2). No intraorbital abscess formation, postseptal extension, or intraocular abnormality was observed. An apparent gaseous shadow overlying the cornea raised concerns regarding a possible gas-forming infection during the initial evaluation. Although definitive characterization was not possible preoperatively, subsequent radiologic review suggested that this finding most likely represented trapped air associated with severe eyelid edema rather than true soft-tissue gas.

Given the rapid progression of symptoms, urgent surgical intervention was planned. Approximately one hour prior to surgery, the patient’s clinical condition deteriorated abruptly. She developed diarrhea, a high-grade fever (40.4 °C), tachycardia (112 beats per minute), and hypotension (systolic blood pressure 79 mmHg; diastolic pressure was unmeasurable). Laboratory testing revealed a C-reactive protein (CRP) level of 29.79 mg/dL, a white blood cell (WBC) count of 16,800/µL, and a creatine kinase level of 399 U/L. Additional blood tests showed a hemoglobin concentration of 11.4 g/dL, serum sodium of 137 mEq/L, serum creatinine of 1.87 mg/dL, and blood glucose level of 103 mg/dL. These findings were consistent with those of STSS. The Laboratory Risk Indicator for Necrotizing Fasciitis (LRINEC) score was 4, classifying the patient as low-risk [11]; however, a rapid test performed on conjunctival swab fluid suggested a group A streptococcal infection. Based on the fulminant clinical course, a diagnosis of periorbital necrotizing soft tissue infection complicated by STSS was made. Emergency eyelid debridement was performed at the bedside in the emergency department, without the use of an operating microscope, following informed consent from the patient.

A skin incision was made along the natural upper eyelid crease, following the double eyelid line, to the level of the tarsal plate, where active bleeding was observed. The layered structure of the orbicularis oculi muscle was preserved. Debridement was limited to the eyelid, as necrosis was restricted to a small focus of subcutaneous adipose tissue. The patient was transferred to the intensive care unit (ICU) for postoperative care.

Empirical systemic antimicrobial therapy consisted of intravenous meropenem (2 g/day), vancomycin (target trough level > 15 µg/mL), and clindamycin (1,800 mg/day). Topical ophthalmic therapy included gatifloxacin (0.3%), tobramycin (0.3%), and cefmenoxime (0.5%) eye drops, administered six times daily. Ofloxacin ophthalmic ointment was applied after irrigation of the eyelid and ocular surface with 50 mL of 0.025% polyvinyl alcohol–iodine (PAI) solution, three times daily, following the confirmation of the absence of iodine hypersensitivity.

**Fig. 3:**
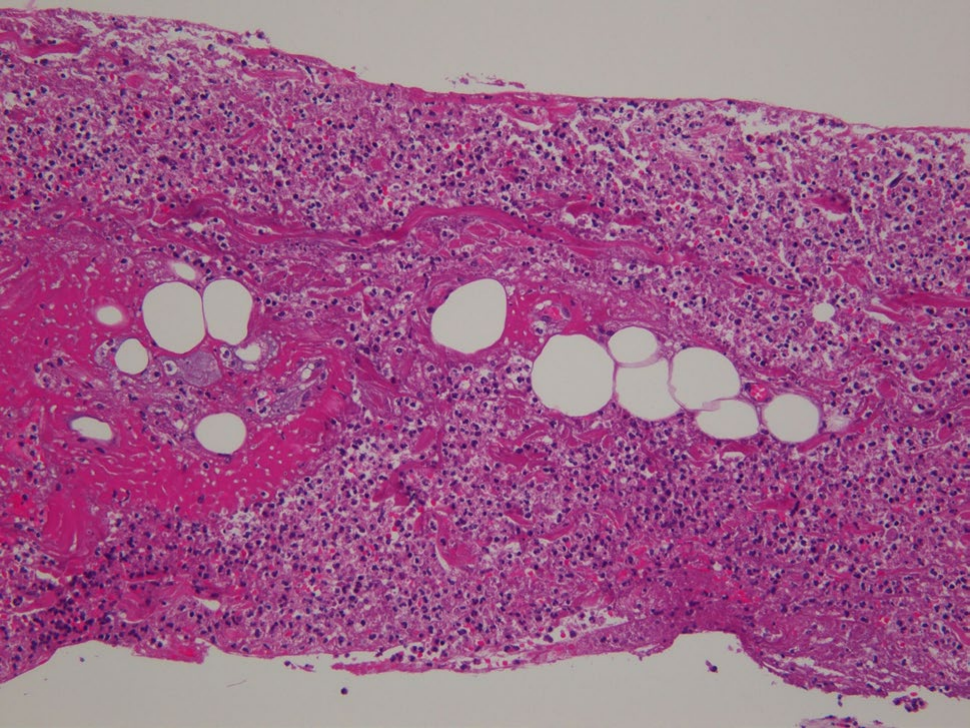
Histopathological examination of tissue removed by debridement. Degenerated and necrotic fibrous connective tissues and adipose tissues infiltrated by numerous neutrophils can be observed

By postoperative day 3, conjunctival hyperemia had not progressed, and no black necrosis of the eyelid was observed (Fig. 1B). Tissue culture from the initial debridement identified *Streptococcus pyogenes* as the sole pathogen. Consequently, meropenem and vancomycin were discontinued and replaced with intravenous ampicillin (8 g/day). Molecular typing identified an M1T strain positive for streptococcal pyrogenic exotoxins (SpE) A and B and negative for SpE C. Histopathological examination revealed necrotic fibroconnective and adipose tissues with marked neutrophilic infiltration (Fig. 3).

Following ICU admission and initiation of intravenous antimicrobial therapy and fluid resuscitation, the patient’s level of consciousness improved. Periorbital and temporal swelling did not progress further. CRP and WBC levels peaked on postoperative day 3 (34.81 mg/dL and 20,210/µL, respectively) and subsequently declined in parallel with clinical improvement.

On hospital day 6, the patient was transferred to the general ward. Increased ocular discharge and recurrence of eyelid swelling were observed (Fig. 1C). Repeat CT imaging revealed no extension into the postseptal or temporal compartments, and microbiological cultures from the debrided site were negative. Consequently, limited bedside debridement was performed twice daily. This procedure involved disinfection of the perilesional skin with a 10% povidone-iodine swab, blunt reopening of the previous incision, and careful removal of small necrotic foci along the muscle plane with sterile swabs until capillary oozing was observed. Adjunctive irrigation with a 0.025% PAI solution was performed for approximately 15 min using a 50 mL syringe, applied from the eyelid to the ocular surface.

The lesion gradually improved without the development of progressive black necrosis. The patient was discharged on hospital day 9 (Fig. 1D). Following her return to her home country, she received oral amoxicillin (1 g three times daily) and clindamycin (450 mg three times daily) for 7 days. A maculopapular rash, attributed to amoxicillin, developed, leading to substitution with cefalexin 500 mg four times daily for 5 days, followed by topical chloramphenicol ointment.

Throughout the clinical course, no visual impairment was observed, and no further surgical interventions were required. Eight months after onset, the right eyelid had completely healed without scarring, preserving both functional and cosmetic outcomes (Fig. 1E and F).

## Discussion

The favorable outcome in this case was likely multifactorial. Early recognition of the rapidly progressive course, prompt initiation of broad-spectrum systemic antibiotics in accordance with international recommendations [12], timely surgical intervention, and intensive care support constituted central components of management [1, 13]. Although the LRINEC score was low [11], clinical deterioration prompted immediate action, suggesting that clinical judgment may outweigh scoring systems in selected cases.

Aggressive surgical debridement remains the cornerstone of treatment for NSTIs [1, 13]. However, in periorbital disease, surgical decisions must balance the eradication of necrotic tissue with preservation of visual function and cosmesis [3–6]. In the present case, necrosis was confined to a small focus of subcutaneous fat, and no postseptal or orbital involvement was identified. Therefore, debridement was intentionally limited to the eyelid, with close postoperative monitoring and readiness to extend the procedure if clinical deterioration occurred. This strategy aligns with selected prior reports describing conservative yet adequate management in localized eyelid disease [8].

Adjunctive irrigation with a low-concentration iodine solution was performed throughout the clinical course. Povidone-iodine is widely used in ophthalmology as a preoperative antiseptic for procedures such as intravitreal injection and cataract surgery [14–16], and dilute concentrations have demonstrated relatively low corneal toxicity [16]. Its use in this case was based on its established antimicrobial spectrum and ocular safety profile, rather than on evidence specific to necrotizing infections. Experimental and wound-healing studies suggest that dilute iodine solutions may exert antimicrobial effects while preserving tissue viability and modulating wound repair pathways [16, 17]. Progressive black necrosis did not develop in this patient. The precise reason for this favorable local outcome remains undetermined; however, immediate initiation of empirical broad-spectrum antimicrobial therapy and timely surgical intervention to preserve perfusion likely played a dominant role. The contribution of adjunctive low-concentration iodine irrigation, if any, is speculative. Nevertheless, considering its safety profile at diluted concentrations and established ophthalmic use, its application in this case was deemed a reasonable adjunct rather than a primary therapeutic determinant.

To conceptualize the structured approach employed in this case, we summarize the key components as “ADAPT” (Table 1). ADAPT is presented as a mnemonic framework reflecting the coordinated and time-sensitive management required in periorbital NSTIs.

**Table 1:** Conceptual summary of the ADAPT framework applied in the present case

Component	Core Concept	Clinical Application in the Present Case
A – Accelerated recognition	Rapid clinical recognition and immediate initiation of empirical broad-spectrum antimicrobial therapy	Fulminant clinical deterioration prompted urgent intravenous broad-spectrum antibiotics despite a low LRINEC score
D – Decisive debridement	Timely incision and removal of necrotic tissue to preserve perfusion	Incision along the natural upper eyelid crease; bleeding confirmed tissue viability; debridement limited to eyelid with readiness to extend if needed
A – Antimicrobial adjustment	Modification of antimicrobial therapy based on microbiological results	Broad-spectrum antibiotics de-escalated to ampicillin after identification of *Streptococcus pyogenes*
P – Periocular antisepsis	Adjunctive local antiseptic support with established ocular safety	Low-concentration iodine irrigation used as supportive measure based on ophthalmic safety data
T – Thorough monitoring	Intensive systemic observation and careful local reassessment	ICU management, serial laboratory evaluation, repeat imaging, and repeat limited bedside debridement when recurrence was suspected

LRINEC: Laboratory Risk Indicator for Necrotizing Fasciitis

ICU: Intensive Care Unit

In conclusion, this case demonstrates that STSS can develop rapidly, even in previously healthy individuals, and that favorable functional and cosmetic outcomes are achievable through early, structured, and multidisciplinary management.

## Data Availability

All relevant data supporting the findings of this case report are included within the article. Additional details are available from the corresponding author upon reasonable request.

## Declarations

### Author informations

S.Y (Shigeko Yashiro): Chief clinician specializing in ocular infectious diseases and inflammatory eye disorders. K.I (Kento Inoue): Specialist in infectious diseases. Y.K (Yuki Kitagawa): Internal medicine resident. K.Y (Kei Yamamoto): Specialist in infectious diseases. Masako N (Masako Nagahara): Clinician specializing in medical retinal disorders. P.V (Phung Vu): Specialist in infectious diseases in Australia. M.U (Mugen Ujiie): Chief of infectious Diseases center. Miyuki N (Miyuki Nagahara): Director of ophthalmic surgery. N.O (Norio Ohmagari): Executive Director of infectious diseases center
